# Design and activity study of a melittin–thanatin hybrid peptide

**DOI:** 10.1186/s13568-019-0739-z

**Published:** 2019-01-30

**Authors:** Xiaofeng Jiang, Kun Qian, Guangping Liu, Laiyu Sun, Guoqing Zhou, Jingfen Li, Xinqiang Fang, Haixia Ge, Zhengbing Lv

**Affiliations:** 1 College of Life Sciences And Medicine, Zhejiang Sci-Tech University, Hangzhou, 310018 Zhejiang China; 2 School of Life Science, Huzhou University, Huzhou, 313000 Zhejiang China; 3 College of Life Sciences, Yan’an University, Yan’an, 716000 Shaanxi China; 4 School of Life Science, Zhejiang University, Hangzhou, 31058 Zhejiang China; 5 Hangzhou Huadihealth Group Ltd. Co., Hangzhou, 31012 Zhejiang China

**Keywords:** Melittin, Thanatin, Hybrid peptide, Structure, Activity

## Abstract

The unique antimicrobial mechanism of antimicrobials make them a promising substitute for antibiotics for fighting drug-resistant bacteria. Both melittin and thanatin have antimicrobial bioactivity. However, thanatin does not inhibit the growth of *Staphylococcus aureus*. Melittin can inhibit *S. aureus* and has strong hemolytic activity. In the present study, the mutant fragments of melittin and thanatin were combined by flexible peptides to form a novel hybrid peptide, which was synthesized based on the secondary and tertiary structure prediction. The hybrid peptide inhibited *S. aureus* with a hemolytic concentration of above 45 μmol/L and inhibition rate in SMMC-7721 cells of 19.14%. The hybrid antimicrobial peptide, which was designed by the combination of α-helix and β-lamellar antimicrobial peptides, showed that both types of peptides did not interact with each either on spatial structure or biological activities, thereby providing a novel idea for the design of artificial antimicrobial peptides.

## Introduction

Generally, the antimicrobial functions of traditional antibiotics are achieved by destroying the bacterial cell wall or blocking the biosynthesis of substances required for the biological activity of bacteria (Goossens et al. 2005; Mangoni and Bhunia 2016). However, the development of antibiotic-resistant bacteria often affects the clinical use of antibiotics. The antimicrobial mechanism of antimicrobial peptides has not been fully understood. Known mechanisms of action include the destruction of cell membranes, interference with nucleic acid and protein synthesis, the inhibition of cell wall synthesis, and interference with cell division (Bolintineanu et al. 2012; Lee and Park 2014; Ursic-Bedoya et al. 2011; Lee et al. 2016; Fabbretti et al. 2012; Malmsten 2014; Cho et al. 2012; Xia et al. 2018). Antimicrobial peptides have unique antimicrobial properties without detectable resistance, making them a promising substitute for traditional antibiotics (Costa et al. 2012; Durrant and Amaro 2015). Aside from determining antimicrobial peptides through biological approaches, researchers often design peptides artificially. Artificial peptides are widely used because of increased antimicrobial spectrum, in vivo stability and biological activity, and reduced cytotoxicity (Wang et al. 2015; Walsh et al. 2011; Godballe et al. 2011).

Thanatin, with a primary structure of GSKKPVPIIYCNRRTGKCQRM, is a type of antimicrobial peptide consisting of 21 amino acid residues. It is found in the insect *Podisus maculiventris*. Despite its broad-spectrum antimicrobial properties, it does not inhibit *S. aureus* (Fehlbaum et al. 1996; Mandard et al. 1998).

Melittin was first obtained from bee venom by Habermann and Jentsch (1967). It is a peptide consisting of 26 amino acids with a primary structure of GIGAVLKVLTTGLPALISWIKRKRQQ. It shows antimicrobial, anti-inflammatory, anti-radiation, anti-arthritic, anti-tumor, anti-AIDS, and other biological activities; It inhibits *S. aureus* (Wachinger et al. 1998; Saini et al. 1999; Gajski and Garajvrhovac 2011). However, its clinical application is limited by its strong toxicity (mainly hemolytic activity), genotoxicity, and influence on gene expression (Gajski et al. 2016; Wu et al. 2015). To improve the biological activity of melittin and reduce its hemolytic activity, many researchers have studied the structure–activity relationship and structural modification of melittin (Blondelle and Houghten 1991; Asthana et al. 2004; Li et al. 2003; Sun et al. 2005).

To expand the antimicrobial spectrum of thanatin and avoid the toxic effects of melittin, a hybrid antimicrobial peptide was designed according to a previous study (Blondelle and Houghten 1991). Hemolysis was slightly affected by the removal of C-terminal amino acids of melittin, according to the previous study (Jahnsen et al. 2015). Four alkaline amino acids (KRKR) are located at the C-terminal amino acids of melittin. The hybrid antimicrobial peptide that includes the full-length of thanatin and the C-terminal of melittin may reveal a stronger and safer antibacterial effect (Leonardo et al. 2012). The hybrid antimicrobial peptide GLPLLISWIKRKRQQ-AGP-GSKKPVPIIYCNRRTGKCQRM was designed using melittin’s C-terminal 15-amino acid mutant GLPL*LISWIKRKRQQ (L*wild type was A) as the N-terminus and thanatin as the C-terminus and by ligating with AGP to create a hybrid peptide that could inhibit *S. aureus* without hemolytic activity. The fusion expression of the hybrid peptide was carried out in *Escherichia coli* by using genetic engineering techniques, and the acid hydrolysis site AP was added to the N-terminus of the hybrid peptide. After the engineered bacteria were fermented, separated, acid-hydrolyzed, and purified, another proline residue was found on the N-terminus of the resulting peptide, that is, PGLPLLISWIKRKRQQGSKKPVPIIYCNRRTGKCQRM. In vitro antimicrobial experiments showed that this hybrid peptide inhibited the growth of *S. aureus*. However, pure hybrid peptide was not obtained due to problems associated with acid hydrolysis. Therefore, the author designed an artificial hybrid peptide (GLPLLISWIKRKRQQGSKKPVPIIYCNRRTGKCQRM) to further explore its antimicrobial, hemolytic, and anti-cancer activities.

## Materials and methods

### Design, physicochemical properties, and structure prediction of polypeptides

The 15 modified amino acid C-terminus of melittin and the complete amino acid sequence of thanatin were ligated using the flexible peptide linker alanine–glycine–proline. Thus, the designed hybrid peptide sequence was GLPLLISWIKRKRQQAGPGSKKPVPIIYCNRRTGKCQRM. By utilizing online tools, the secondary and tertiary structure, molecular weight, and isoelectric point of the original peptides and the designed hybrid thanatin and melittin peptide were predicted. Secondary structure prediction was based on GOR (http://npsa-Pbil.ibcp.fr/cgi-bin/npsa_automat.pl?page = npsa_gor4.html) and the HNN method (https://prabi.ibcp.fr/htm/site/web/home), and the advanced structure was predicted based onPhyre^2^ (http://www.sbg.bio.ic.ac.uk/phyre2/html) and SWISS-MODEL (https://swissmodel.expasy.org/) programs.

### Polypeptide biosynthesis

The peptide was synthesized from the C-terminus to N-terminus end by the solid-phase synthesis method (ChinaPeptides Co., Ltd.). The synthesized peptide chain was oxidized with dimethyl sulfoxide (DMSO) to form a ring, after which it was purified by high performance liquid chromatography, and freeze-dried into powder with a purity of 98.06%.

### Bacteria

*Escherichia coli* JM109 strain was obtained from the School of Life Science, Huzhou Univesity. *S. aureus* (1.282), *Bacillus subtilis* (1.15792) and *Salmonella typhimurium* (1.1190) were purchased from China General Microbiological Culture Collection Center (CGMCC). The sterile defibrinated sheep blood was obtained from Pingrui Biotechnology (Beijing) Co., Ltd.

### Antibacterial test

*Escherichia coli* JM109, *S. aureus*, *B. subtilis*, and *S. typhimurium* were inoculated into liquid PB medium (1% peptone and 0.9% sodium chloride) at 37 °C and 180 rpm for 24 h, and the PB medium was diluted to 300 bacteria/80 μL. The hybrid antimicrobial peptide was diluted with PB medium into 1.5, 3, 6, 12.5, 25, 50, 100, 200, 400, and 12 μmol/L. Twenty microliters of the hybrid antimicrobial peptide solution was added to a 96-well plate, and then 80 μL of the diluted bacterial solution was added for a total of 100 μL. A positive control (PB medium + ampicillin) and a negative control (PB medium only) were also included. The 96-well plates were incubated for 12 h at 25 °C with slow shaking (~ 100 rpm) (Taguchi et al. 2000).

### Hemolysis test

Two milliliters of defibrinated sheep blood were obtained and centrifuged at 2000 rpm for 10 min, and the pellet was kept and washed with normal saline until no blood color remained. Next, saline was added and diluted to 2% of the red blood cell suspension. A volume of 2.5 mL of the red blood cell suspension was added into seven test tubes. Then, 2.5 mL of increasing concentrations of the antimicrobial peptide (final concentration: 5, 15, 30, 45, and 60) was also added to each tube, whereas 2.5 mL of normal saline and 2.5 mL of distilled water were used as a negative and positive controls, respectively. After homogenization, the tubes were incubated in a water bath at 37 °C and observed every 15 min for the first hour and once an hour for the next 3 h (Wei et al. 2010).

### Anticancer tests

These tests took three experimental groups of the culture medium, control, and test group (the concentration of hybrid peptide was 100 µg/mL). Each group contains 3 double-wells and was repeated thrice. After the preserved SMMC-7721 cell line (BNCC338089, purchased from Bnbio company, Beijing, China) were reactivated by the RPMI-1640 medium and were cultured to the logarithmic phase, the cells were washed by phosphate buffer (pH 7.3) thrice. Then, the cells were digested with 0.25% of trypsin–EDTA-2Na for 2–3 min. Digestion was terminated by the cultured medium, and the cell suspension was transferred into the centrifuge tube. After 3 min of centrifugation (1000 rpm), the precipitated cells were resuspended by the RPMI-1640 medium and then diluted to 2.5 × 10^4^ cells/mL. All cell plates were covered with board. The culture medium group was mixed with 200 µL of the RPMI-1640 medium, and the control and test group were mixed with 100 µL of cell suspension. Then, each group was incubated in 5% CO_2_ and saturation vapor incubator for 3–4 h at 37 °C. A total of 100 µL of the RPMI-1640 medium was added into the control group, and 100 µL of hybrid peptide was added into the test group after incubation. The culture was continued for 44 h. The medium was absorbed, and each plate was washed with phosphate buffer. After washing, 100 µL of phosphate buffer and 20 µL of MTT were added into each group. After 4 h, incubation was completed, and the media were consumed. A volume of 100 µL of DMSO was then added into each hole. After 10 min of low-speed shock, the absorbance (A490 mm) values were measured using the microplate reader.

## Results

### Physicochemical properties and structure prediction of polypeptides

#### Physicochemical properties of the hybrid peptide

The isoelectric point and molecular weight of the designed hybrid peptide are shown in Table 1 (thanatin data from the reference) (Fehlbaum et al. 1996).

**Table 1:** Physicochemical properties of hybrid peptide

Peptides	Isoelectric point (pI)	Molecular weight (Da)
Melittin Peptide	12.02	1836.26
Thanatin	10.48	2436.20
Hybrid Peptide	11.50	4576.56

#### Secondary structure prediction by the GOR method

The secondary structure of the modified C-terminal amino acid sequence of melittin was predicted to be: ccccchhhhhcccee, where **h** represents alpha helix, **c** represents random coil, and **e** represents extended strand.

The predicted secondary structure of thanatin was: ccccceeeeeecccccccccc.

Therefore, as anticipated, the predicted secondary structure of the hybrid peptide was: ccccchhhhhhhhhccccccccceeeeeeccccccccee.

#### Secondary structure prediction by the HNN method

The secondary structure of the modified C-terminal amino acids of melittin based on the HNN method are as follows:

DSC ccchhhhhhhhhccc

MLRC ceeeeeeeeeeeeec

PHD ccceceehhhhhhhc

Sec.Cons. ccce?eehhhhh??c

The predicted secondary structure of thanatin is as follows:

DSC cccccceeeeecccccccccc

MLRC ccccceeeeeeeccccccccc

PHD ccccccceeeecccccccccc

Sec.Cons. cccccceeeeecccccccccc

The predicted secondary structure of the hybrid peptide is as follows:

DSC cccchhhhhhhhhhcccccccccceeeeecccccccccc

MLRC ccchhhhhhhhhhhcccccccccceeeeeeccccccccc

PHD cceeeeeehhhhhhhcccccccceeeeeeeccccccccc

Sec.Cons. ccc?hhhhhhhhhhcccccccccceeeeeeccccccccc

### Advanced structural prediction

**Fig. 1:**
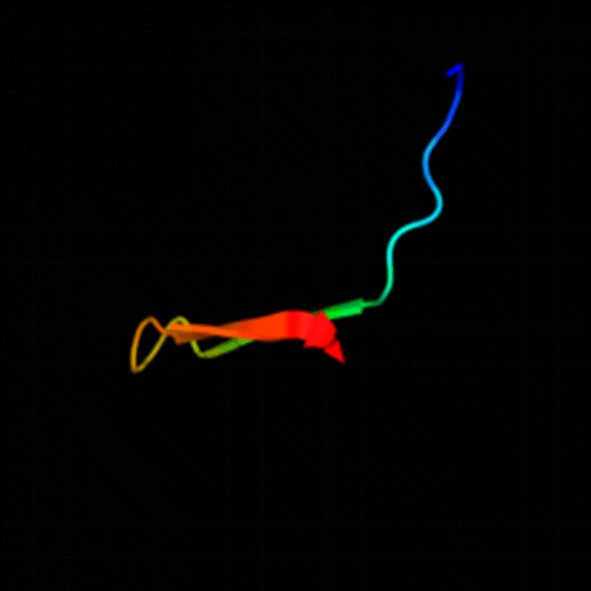
Phyre^2^ predicted tertiary structure of the hybrid peptide (arrow from N C)

**Fig. 2:**
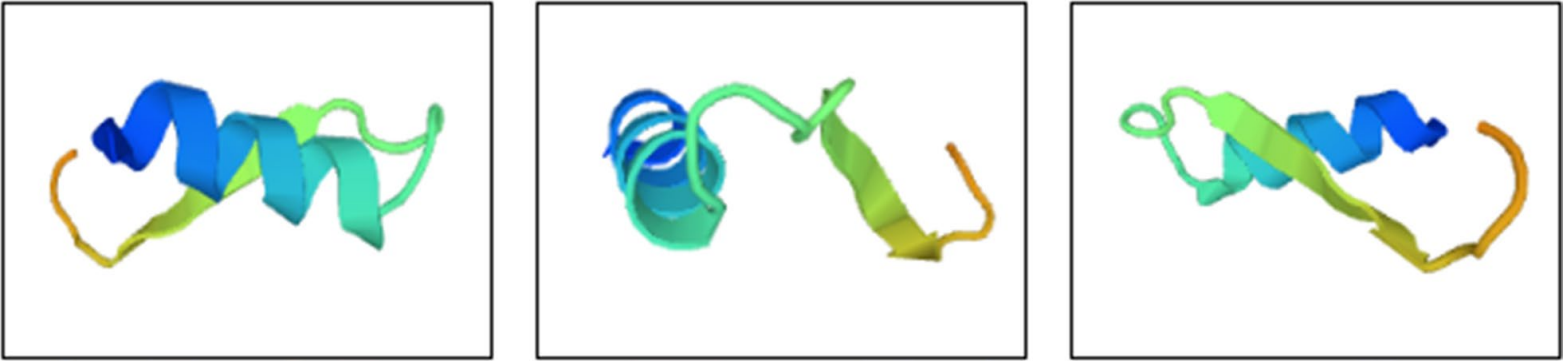
SWISS-MODEL prediction tertiary structures

To further investigate the hybrid peptide structure, we determined the predicted tertiary structure by using two methods. First, Phyre^2^ was used in our research. The tertiary structure prediction based on Phyre^2^ is shown in Fig. 1. The second method utilized for predicting the tertiary structure of the hybrid peptide was SWISS-MODEL. Seven similar templates were predicted with a consistency of 31.03. The spatial simulation structure is shown in Fig. 2.

### Antibacterial test

The minimum inhibitory concentration (MIC) of the hybrid antimicrobial peptide and melittin on *E. coli*, *S. aureus*, *B. subtilis and S. typhimurium* were detected and listed in Table 2.

**Table 2:** Minimum inhibitory concentration (MIC) range of antimicrobial peptides

Bacterial name	Inhibitory concentration range (hybrid)	Inhibitory concentration range (melittin)
*Escherichia coli* JM 109	1.2–2.5 μmol/L	0.6–1.2 μmol/L
*Staphylococcus aureus*	1.2–2.5 μmol/L	0.9–1.5 μmol/L
*Bacillus subtilis*	2.5–5 μmol/L	0.6–1.2 μmol/L
*Salmonella typhimurium*	1.2–2.5 μmol/L	0.3–0.6 μmol/L

### MIC and hemolysis test

Functional evaluation of the hybrid peptide and melittin was determined by establishing the minimum inhibitory concentration (MIC) and by performing a hemolysis test. The MIC of the hybrid antimicrobial peptide against *E. coli* JM109, *S. aureus*, *B. subtilis*, and *S. typhimurium* is shown in Table 2.

Hemolysis test performed using the hybrid peptide showed that hemolysis did not occur at 45 μmol/L but rather at 60 μmol/L, indicating that the hemolytic concentration of the hybrid antimicrobial peptide was greater than 45 μmol/L.

### Anticancer tests

The result of the mean difference analysis showed a significant difference between the control and test groups (*P *< 0.05), and the average inhibitory rate of the hybrid peptide in the SMMC-7721 cells was 19.14%.

## Discussion

Protein structural modification and the antimicrobial peptides have always been a research hotspot, as well as the antimicrobial peptides. The modification of various antimicrobial peptide structures (Gao and Zhu 2012; Wang et al. 2015), the splicing of different peptides (Acuña et al. 2012; Kim et al. 2016; Memariani et al. 2016; Orrapin and Intorasoot 2014), antimicrobial peptides incorporating non-natural amino acids, or artificial and computer-aided design of antimicrobial peptides (Wang et al. 2016) are all common means of peptide modifications. Melittin’s hemolytic activity has limited its clinical application, prompting researchers to study the causes of hemolysis and modify it (Wu et al. 2016; Sun et al. 2005; Yan et al. 2003; Juvvadi et al. 2010).

In the present study, the hybrid peptide was designed by using melittin’s C-terminal 15-amino acid mutant (with weak hemolytic activity) (Li et al. 2003) as the N-terminus and thanatin as the C-terminus. Based on the secondary structure prediction by using the GRO method, the secondary structure of the hybrid peptide’s C-terminus was not changed compared with thanatin. However, the N-terminus showed an increased helicity compared with the mutant fragments of melittin. From the secondary structure prediction by using the HNN method, the secondary structure of the hybrid peptide’s C-terminus was not changed compared with thanatin. The N-terminal helicity was not reduced compared with the mutant fragments of melittin. The melittin fragment and thanatin contained less than 30 amino acid residues, thereby contributing to the uncertainty over the tertiary structure prediction. Hence, the spatial structure of the three peptides cannot be compared. From the tertiary structure of the hybrid peptide model generated by the Phyre^2^ and SWISS-MODEL, the N-terminal melittin fragment had an α-helix structure, whereas the C-terminal thanatin was composed of three independent structures, including random peptide fragments, β-lamella, and random peptide fragments. Considering the presence of independent α-helix and β-lamellar structures, the hybrid peptide retained the ability to inhibit *S. aureus*. The MIC of the hybrid peptide on *S. aureus* and *B. subtilis* was nearly consistent with that of the melittin fragment and thanatin, and its MIC on *E. coli* JM109 and *S. typhi* was also increased (Fehlbaum et al. 1996; Li et al. 2003; Taguchi et al. 2000). These results suggested that the melittin fragment and thanatin almost did not interact with each another on the spatial structure of the hybrid peptide, and each peptide fragment retained its original antimicrobial properties. The retention of the melittin fragment’s activity might contribute to the anti-cancer activity of the hybrid peptide. In this study, a hybrid antimicrobial peptide was designed by the combination of α-helix and β-lamellar antimicrobial peptides. Both types of peptide did not interact with each other either in terms of spatial structure or biological activities, thereby providing ideas for the design of artificial antimicrobial peptides.
